# *LHFPL5* splice site variant in a cat with deafness and vestibular dysfunction

**DOI:** 10.1002/age.70062

**Published:** 2025-12-16

**Authors:** Assami‐Carina Perret, Julien Guevar, Vidhya Jagannathan, Tosso Leeb

**Affiliations:** ^1^ Institute of Genetics, Vetsuisse Faculty University of Bern Bern Switzerland; ^2^ Clinique Vetelys Geneva Switzerland

**Keywords:** animal model, *Felis catus*, inner ear, neurology, precision medicine, veterinary medicine

## Abstract

Vestibular disorders associated with hearing loss are indicative of inner ear dysfunction. We investigated a young cat presenting with deafness and vestibular signs. Magnetic resonance imaging of the brain revealed no abnormalities. No obvious visual impairment was reported on ophthalmological examination. Whole‐genome sequencing of the affected cat and comparison with 106 control genomes identified a private homozygous splice site variant in the *LHFPL5* gene, XM_003986102.4:c.413‐2A>G. In humans, *LHFPL5* variants are known to cause autosomal recessive deafness, sometimes accompanied by bilateral vestibular areflexia. The LHFPL5 protein is essential for hearing and balance, as it anchors the tip link of inner ear hair cells to the mechano‐electrical transducer channel. The identified splice site variant in the investigated cat is likely to result in loss of functional LHFPL5 and represents a candidate causal variant for the observed auditory and vestibular dysfunction in the affected cat.

Deafness is sometimes associated with vestibular dysfunction due to the shared embryological origin and anatomical proximity of auditory and vestibular structures in the inner ear (Rossmeisl, 2010; Ryugo & Menotti‐Raymond, 2012; Seiwerth, 2023; Thomas, 2000). Studies in humans have shown that children with congenital deafness frequently present vestibular deficits, which may affect motor development (Dy et al., 2022; Shinjo et al., 2007).

In humans, more than 50% of congenital hearing impairments are hereditary. The remainder are due to environmental causes such as infection, trauma, excessive noise exposure, or ototoxicity. Among the inherited forms, isolated or non‐syndromic hearing impairment disorders account for about 70% and most of them represent monogenic disorders. More than 80 candidate genes for early onset sensorineural deafness have been identified (Petit et al., 2023; Strain, 2015).

A 30‐month‐old cat was presented with a chronic history of deafness, vestibular signs, intermittent aggressive behavior, and vocalizations. The owners reported that the cat had never shown any normal response to noise or calling. According to the owners, the affected cat had an unaffected sibling with normal reactions to noise and no clinical signs compatible with neurological or visual impairment. The unaffected sibling was not available for further investigations. The affected cat had reportedly been previously exposed to toxoplasmosis (elevated IgG and normal IgM) and treated with clindamycin. Neurological examination identified loud vocalizations while pacing into the room, which were subjectively judged to be related to hearing impairment rather than pain. A bilateral head swaying movement was also observed together with a low head and body posture close to the ground (Video S1). An ophthalmological examination was unremarkable.

The suspected localization was at the level of the vestibular and cochlear systems. A degenerative or inflammatory condition was suspected, although a malformation could not be ruled out.

Magnetic resonance imaging of the brain did not reveal any abnormalities. Urine organic acids were analyzed, and several hereditary metabolic disorders were ruled out, including methylmalonic aciduria, malonic aciduria, L‐2‐hydroxyglutaric aciduria, type I and II hyperoxaluria, and glyceric aciduria. No recording of brainstem auditory evoked potentials was performed. The cat was euthanized at age 42 months due to persistent loud vocalizations and owner uncertainty whether the cat was experiencing pain.

Given the early age of onset and absence of any lesion on magnetic resonance imaging, an underlying genetic defect was suspected. We therefore obtained an EDTA blood sample from the affected cat and extracted genomic DNA. A PCR‐free library was prepared and whole genome sequencing was performed at 20× coverage on an Illumina Novaseq 6000 instrument. Mapping, variant calling with respect to the F.catus_Fca126_mat1.0 reference genome assembly, and variant annotation using NCBI annotation release 105 were performed as described (Jagannathan et al., 2019).

Comparing the sequencing data to 106 feline control genomes resulted in 443 heterozygous and 53 homozygous private protein changing variants (Tables S1 and S2).

Among these were two homozygous missense variants in *ADGRV1*, XP_044889500.1:p.(Asn5140Lys) and XP_044889500.1:p.(Ala5527Thr). Variants in *ADGRV1* may cause Usher syndrome type 2C in human patients, which is characterized by normal vestibular function, mild congenital hearing loss that progresses with age, and progressive retinitis pigmentosa (OMIM #605472; Weston et al., 2004). As the affected cat had a profound deafness phenotype with vestibular dysfunction from birth and as the ophthalmological examination had not shown any loss of vision or signs of retinal degeneration, the *ADGRV1* variants were not further investigated.

Additionally, a private homozygous splice site variant was identified in *LHFPL5*. This variant, NC_058372.1:g.34339237A>G or NC_058372.1(XM_003986102.4):c.413‐2A>G affects the conserved splice acceptor site of the second coding exon. This variant is predicted to disrupt normal mRNA splicing, probably resulting in loss of function of the *LHFPL5* gene (Figure 1).

**FIGURE 1:**
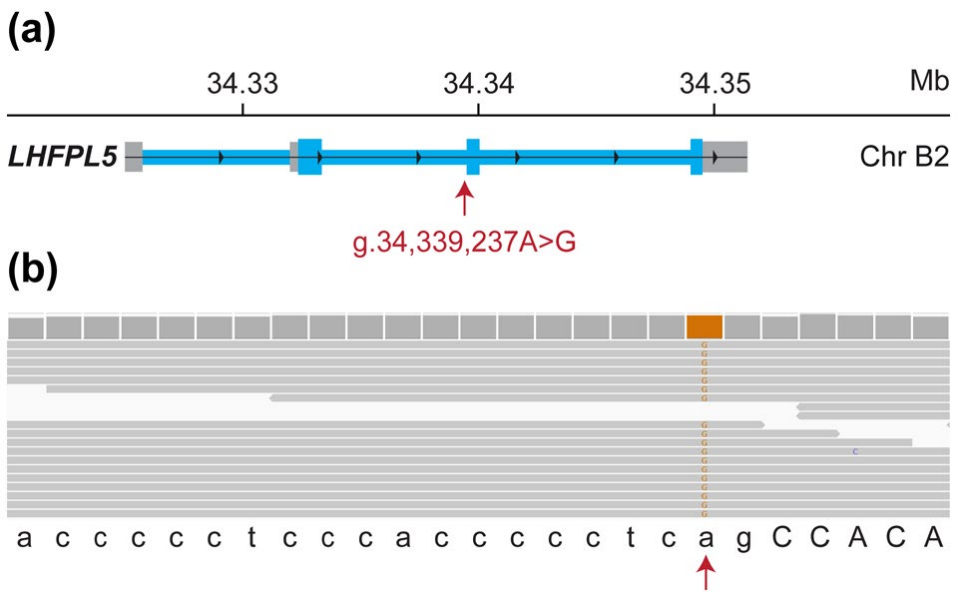
Details of the *LHFPL5* splice site variant. (a) Genomic organization of the *LHFPL5* gene and position of the variant. (b) An Integrative Genomics Viewer screenshot of the short‐read alignments illustrates the variant. The reference sequence is indicated at the bottom. Intronic bases are in lowercase letters, exonic bases in uppercase letters. The NC_058372.1:G.34339237A>G variant affects the conserved splice site at the end of intron 2 (c.413‐2A>G).

*LHFPL5* encodes LHFPL tetraspan subfamily member 5, a protein with four transmembrane domains that is expressed in cochlear hair cells, which are essential for hearing and balance. Incoming sound is registered as mechanical force on the tip links joining adjacent stereocilia on the surface of hair cells. This mechanical force is then converted into an ion current by the mechano‐electrical transducer (MET) channel in the membrane of hair cells. LHFPL5 physically connects the tip link to the MET and helps to anchor the MET correctly within the membrane. This protein interaction triggers the MET channel to open in response to mechanical force sensed by the tip links. A specific region of LHFPL5 directly interacts with an amphipathic helix in the TMC1 protein, a key component of the MET channel. When this interaction is disrupted, the channel no longer responds properly to mechanical stimuli (Beurg et al., 2024; Qiu et al., 2023).

Several variants in *LHFPL5* have been identified in human patients with autosomal recessive congenital non‐syndromic deafness (DFNB67; OMIM #610265; Shabbir et al., 2006; Kalay et al., 2006; Lerat et al., 2019). The phenotype observed in humans is characterized by severe bilateral hearing loss with or without vestibular abnormalities. In some young patients, bilateral vestibular areflexia has been reported, with a mean age of walking acquisition at 19 months (Lerat et al., 2019). In mice, a spontaneously arisen mutant termed hurry‐scurry (*hscy*) was described, which is characterized by profound deafness, circling behavior, frequent head shaking, and an inability to swim suggestive of vestibular dysfunction. The *hscy* phenotype is inherited as an autosomal recessive trait and caused by an *Lhflp5*:p.C161F missense variant (Longo‐Guess et al., 2005). The phenotype in the investigated cat closely resembled the phenotypes observed in human DFNB67 patients and *hscy* mice. To the best of our knowledge, we report the first domestic animal with *LHFPL5* loss of function.

We acknowledge several limitations of our study. The investigated cat was a single, non‐pedigreed individual, which precluded the analysis of related cases and analysis of genotype–phenotype co‐segregation. Moreover, as the cat was euthanized at a relatively young age, early stages of retinitis pigmentosa without gross vision impairment might have been missed. Therefore, although we consider that the variants identified in the *AGDR1V* gene are unlikely to be causative, we cannot definitively exclude the possibility that one or both has a functional role and may also have contributed to the phenotype. Applying the ACMG/AMP criteria for the interpretation of sequence variants in human diagnostics (Richards et al., 2015), due to the limited available evidence, the *LHFPL5* and the two *AGDR1V* variants would have to be classified as variants of uncertain significance.

In conclusion, we consider the *LHFPL5*:c.413‐2A>G splice site variant to be the most likely candidate causative variant. This is the first report of a potential *LHFPL5*‐related inherited deafness in a domestic animal.

## AUTHOR CONTRIBUTIONS

**Assami‐Carina Perret:** Investigation; writing – original draft; writing – review and editing. **Julien Guevar:** Conceptualization; investigation; writing – original draft; writing – review and editing. **Vidhya Jagannathan:** Writing – review and editing; data curation. **Tosso Leeb:** Conceptualization; writing – original draft; writing – review and editing; supervision.

## CONFLICT OF INTEREST STATEMENT

The authors declare no conflicts of interest.

## ETHICS STATEMENT

The cat in this study was privately owned and the sample was collected with the consent of its owner. Sampling of the affected cat was performed for diagnostic or therapeutic reasons and did not constitute an animal experiment in the legal sense. The collection of blood samples from control cats was approved by the “Cantonal Committee For Animal Experiments” (Canton of Bern; permit BE94/2022; Approval date: 30‐11‐2022).

## Supporting information


Table S1.

Table S2.

Video S1.

## Data Availability

All data are freely available. Accessions for the whole genome sequence data are given in Table S1.
